# Pharmacogenomic Considerations for Anticoagulant Prescription in Patients with Hereditary Haemorrhagic Telangiectasia

**DOI:** 10.3390/jcm12247710

**Published:** 2023-12-15

**Authors:** Sarah C. McCarley, Daniel A. Murphy, Jack Thompson, Claire L. Shovlin

**Affiliations:** 1 National Heart and Lung Institute, Imperial College London, London W12 0NN, UK; sarah.mccarley22@imperial.ac.uk (S.C.M.); jack.thompson7@nhs.net (J.T.); 2 Pharmacy Department, Imperial College Healthcare NHS Trust, London W2 1NY, UK; daniel.murphy10@nhs.net; 3 Social, Genetic and Environmental Determinants of Health Theme, NIHR Imperial Biomedical Research Centre, London W2 1NY, UK; 4 Specialist Medicine, Hammersmith Hospital, Imperial College Healthcare NHS Trust, London W12 0HS, UK

**Keywords:** anticoagulation, direct oral anticoagulant, pharmacogenomics, loss-of-function variant, missense variant, genetic testing

## Abstract

Hereditary haemorrhagic telangiectasia (HHT) is a vascular dysplasia that commonly results in bleeding but with frequent indications for therapeutic anticoagulation. Our aims were to advance the understanding of drug-specific intolerance and evaluate if there was an indication for pharmacogenomic testing. Genes encoding proteins involved in the absorption, distribution, metabolism, and excretion of warfarin, heparin, and direct oral anticoagulants (DOACs) apixaban, rivaroxaban, edoxaban, and dabigatran were identified and examined. Linkage disequilibrium with HHT genes was excluded, before variants within these genes were examined following whole genome sequencing of general and HHT populations. The 44 genes identified included 5/17 actionable pharmacogenes with guidelines. The 76,156 participants in the Genome Aggregation Database v3.1.2 had 28,446 variants, including 9668 missense substitutions and 1076 predicted loss-of-function (frameshift, nonsense, and consensus splice site) variants, i.e., approximately 1 in 7.9 individuals had a missense substitution, and 1 in 71 had a loss-of-function variant. Focusing on the 17 genes relevant to usually preferred DOACs, similar variant profiles were identified in HHT patients. With HHT patients at particular risk of haemorrhage when undergoing anticoagulant treatment, we explore how pre-emptive pharmacogenomic testing, alongside HHT gene testing, may prove beneficial in reducing the risk of bleeding and conclude that HHT patients are well placed to be at the vanguard of personalised prescribing.

## 1. Introduction

Hereditary haemorrhagic telangiectasia (HHT) is an autosomal dominant multisystem vascular dysplasia arising from a single heterozygous loss-of-function variant (“mutation”), usually in *ENG*, *ACVRL1*, or *SMAD4* [1,2,3,4,5]. As recently reviewed [6,7,8,9], patients develop internal, visceral arteriovenous malformations (AVMs) and smaller telangiectasia that bleed recurrently. International consensus is available to guide clinical management [7,8,10,11]. Initial guidance was through the generation of consensus clinical diagnostic criteria (the Curaçao Criteria) where the presence of three criteria from spontaneous recurrent nosebleeds, mucocutaneous telangiectasia, visceral involvement, and family history can be used to define definite clinical HHT [10,11,12]. These criteria are less helpful in children where there are fewer clinical features [9,13,14,15], and conversely, it is possible to overdiagnose HHT if based on nosebleeds, telangiectasia, and family history alone [16,17]. The 2020 Second International Guidelines [7] recommended obtaining a genetic diagnosis of the HHT-causative mutation to facilitate targeted screening for internal AVMs. HHT gene testing pathways are now in place in multiple countries worldwide [7,8,18,19,20,21,22,23,24,25] and facilitate the diagnosis of HHT, targeted AVM screening programmes, and direction of *SMAD4* families to *SMAD4*-specific preventative measures [26,27,28,29,30,31].

Haemorrhage and anemia are hallmarks of HHT. Recurrent bleeds from nasal and/or gastrointestinal telangiectasia in HHT commonly result in anaemia and dependence on oral iron, intravenous iron, and/or red cell transfusions [7,8,32,33], with anaemia exacerbated by additional aetiologies [34,35,36,37]. Despite this, there are frequent indications for therapeutic anticoagulation. One major indication is venous thromboembolism (VTE), which is more common in HHT than in the general population [38,39,40] and may also be precipitated by therapies to treat HHT bleeding [41,42]. A further common indication is atrial fibrillation, which complicates high cardiac output states in the setting of HHT hepatic AVMs, particularly when anaemia develops [43]. Left atrial appendage closure is increasingly performed for HHT patients unable to tolerate the usually recommended anticoagulation [44,45,46]. In addition to treatment (therapeutic) doses, anticoagulants are often indicated at lower (prophylactic) doses, for example, peri-operatively or in hospitalized, acutely ill medical patients [47,48].

Previous observational data in HHT-affected individuals have indicated marked extremes in tolerance of anticoagulant therapies. HHT is the second most common heritable bleeding disorder after von Willebrand’s disease (HHT 11–17 per 100,000 [49,50,51,52]; VWD 109–2200 per 100,000 [53]) as it is more common than the haemophilias [54]. Given that up to 10,000 HHT patients in Europe are estimated to require anticoagulation [55], it is surprising that a modest number of small retrospective cohort studies (Supplementary Table S1) represent the bulk of our knowledge on the safety and tolerability of different anticoagulant drugs in HHT [39,40,55,56,57,58,59,60]. Incidence of major bleeding episodes necessitating the discontinuation of anticoagulation ranged from 21.6 to 50.1 per 100 patients per year across studies [39,58]. For example, a large retrospective cohort study surveying 126 patients on the French national HHT registry found that just over a third of HHT patients prematurely discontinued anticoagulation in the first three months of treatment [60]. Reasons for discontinuation included mucosal bleeding, major bleeding events, and corresponding increases in red cell transfusions and/or hospitalisation [60].

Until recently, it was not possible to directly compare anticoagulant agents in HHT as no single study included the full range of low molecular weight heparins (LMWH), Vitamin K antagonists such as warfarin/acenocoumarol, and direct oral anticoagulants (DOACs) in any significant numbers (Supplementary Table S1). In part, this reflected the reluctance of major HHT centres to prescribe DOACs where there is less opportunity to reverse, and no evidence of tolerance in HHT, in contrast to heparin and warfarin [56,57]. For instance, in early data from the Hospital Italiano de Buenos Aires, no DOAC use was reported in their HHT registry [61], while across Europe, only 32 DOAC treatment episodes were identified by the European Reference Network [55]. Comparing these small numbers to historical online patient survey data [57] and expert opinion led the Second International Guidelines Committee to suggest LMWH and warfarin over DOACs in 2020 [7]. More recently, US data provided sufficient numbers to enable direct comparisons between anticoagulant agents in HHT patients for the first time [59]. This study found that the rates of dose-reduction or premature treatment discontinuation due to bleeding were similar in those episodes involving warfarin (16/35 [46%]), heparin-based anticoagulation (LMWH or fondaparinux, 14/27 [48%]), DOACs (11/25 [44%]), and multiple agents simultaneously (18/41 [44%]), noting that anticoagulation discontinuation rates were potentially higher than reported in some earlier studies [40,55,56]. In view of variability, missing data, and some analyses that combined prophylactic (low dose) and therapeutic episodes (Supplementary Table S1), despite identifying 356 anticoagulant treatment episodes in HHT across 13 cohort studies and 64 case reports, the authors of a 2023 “scoping review” did not feel they could make over-arching conclusions on the optimal anticoagulation agent in HHT [62].

What these studies do show is that while more people with HHT tolerate therapeutic anticoagulation with no discernible adverse consequences [39,40,55,56,57,58,59,60,61,62], a significant proportion of patients on heparin, Vitamin K antagonists, and DOACs such as apixaban, dabigatran, edoxaban, and rivaroxaban, have serious exacerbation in their bleeding diathesis and have to discontinue therapy [39,40,55,56,57,58,59,60,61,62]. Crucially, several series demonstrate that tolerance differs between different anticoagulant agents in the same patient [55,57].

There is no evidence in man that a specific HHT causal genotype is associated with tolerance of individual anticoagulant agents [55] or, indeed, a higher or lower overall HHT bleeding risk [4,63]. This is supported by recent data from mice that indicate that in both major HHT genotypes (*ACVRL1* and *ENG)*, haemostasis is impaired to a similar degree, though through different mechanisms [64]. Thus, simple HHT diagnosis with or without HHT gene testing does not allow prediction of who is at higher risk of bleeding, or higher risk of bleeding on prescription of anticoagulants. Beyond HHT-causal genes, however, whole genome sequencing (WGS) data in 104 HHT patients recruited to the 100,000 Genomes Project demonstrated that patients with greater haemorrhagic severity had more deleterious variants in genes encoding platelet and coagulation cascade-related proteins [63].

These observations prompted us to extend genomic analyses to additional genes that may modify the HHT bleeding phenotype, focusing on commonly prescribed drugs. The field of pharmacogenomics examines the impact of variation in the genome on drug pharmacology and offers the potential to reduce adverse events and improve drug efficacy [65]. Loss and gain-of-function alleles in multiple genes have been shown to affect drug pharmacokinetics (how the body handles drugs) and pharmacodynamics (how drugs affect the body) [66]. Recent guidelines for the general population detail multiple genes with DNA variants that have sufficient clinical impact to merit changes to the drug or dose [67,68,69]. In the general population, a major goal is to reduce adverse events and hospital admissions, and for anticoagulants, the greatest concern is major bleeding events [70]. These concerns are exacerbated for people with HHT, who are already prone to bleeds due to abnormal vascular structures. Separately, there are pharmacogenomic considerations for efficacy, in other words, preventing pathological thromboses. While that is less of a focus for the current study, the higher VTE rates in HHT mean that efficacy considerations within what may be a narrower therapeutic window are also important.

Therefore, we considered pharmacogenomic considerations of anticoagulant therapies to be particularly relevant for people with HHT. Our first goal was to provide evidence to support or refute individual drug-specific intolerance so that a single failed anticoagulation episode does not prevent future use of other anticoagulants. A second goal, focusing on countries where gene testing is implemented in HHT diagnostics, was to evaluate if there could be an indication for diagnostic pharmacogenomic testing in HHT, using linkage disequilibrium and pharmacogenetic DNA variant prevalence in general and HHT populations. We specifically focused on the newer DOACs that are favoured in general population guidelines and by patients but where more extreme haemorrhagic responses have been described in HHT [55].

## 2. Materials and Methods

### 2.1. Gene Identification

A literature search was conducted to reconstruct the warfarin, heparin, and DOAC biochemical pathways and underlying genes. The search was conducted using the Google Scholar and Pub Med databases. The search included key phrases such as “Anticoagulants AND Pharmacogenetics”, “Pharmacogenetics AND Warfarin”, “Warfarin metabolism”, “Warfarin biochemical pathway”, “Pharmacogenetics AND Heparin”, “Heparin metabolism”, “Heparin biochemical pathway”, “Pharmacogenetics AND DOACs”, “DOACs metabolism”, and “DOACs biochemical pathway”. Articles on each anticoagulant biochemical pathway and anticoagulant pharmacogenetics were selected. The Data Supplement provides a detailed appreciation of gene involvement in the anticoagulants’ biochemical pathways.

In a separate study (Murphy et al., manuscript in preparation), the 17 genes with actionable guidance in clinical practice were generated by making use of the Dutch Pharmacogenetics Working Group [71] and the Clinical Pharmacogenetics Implementation Consortium [72] clinical guidelines, each derived from data within the Pharmacogenomics Knowledgebase (PharmGKB) [73].

### 2.2. Gene Location and Variant Identification

Gene positions on the Genome Reference Consortium human(GRCh) build 38 [74] were identified using the University of California Santa Cruz (UCSC) Genome Browser [75]. RefSeq [76] was used to obtain further metrics for each gene and major transcripts.

Data from 76,156 participants in the Genome Aggregation Database (gnomAD) v3.1.2 [77] were used to estimate general population variant prevalence, specifically for predicted loss-of-function (pLOF) variants (nonsense, frameshift, and consensus splice site variants), and separately for missense variants that result in an amino acid substitution.

Anonymised outputs from the Genomics England internal bioinformatics pipelines and analyses were examined in the Genomics England Research Environment [78] using Participant Explorer and Interactive Variant Analysis (IVA) v2.2.3. Data were approved for export through the Research Environment AirLock under subproject RR42 (HHT-Gene-Stop).

### 2.3. Data Analysis

A dataset was constructed to visualise the variation in the pharmacogenes using publicly available databases. GRCh38 [74] and MANE Project v1.2 [79] were used to obtain the length of each gene, the length of the coding region in nucleotides, and the number of exons. Within gnomAD 3.1.2 [77], for each pharmacogene, we extracted the total number of missense and pLOF variants (separately and combined), the total number of individuals reported each missense or pLOF variant, and the allele frequency of each variant. The number of gene variants per coding region nucleotide was calculated.

Data from HHT patients recruited to the 100,000 Genomes Project [78] were used to construct a dataset containing the total number of variants and pLOF variants in the HHT cohort for each of the DOAC pharmacogenes. We then calculated the number of variants per [HHT] individual, pLOF variants per [HHT] individual, variants in an individual per coding sequence nucleotide, and the ratio of the number of variants in an HHT individual to the number of variants in the general population per coding sequence for each of the DOAC pharmacogenes.

Descriptive, comparative, and relationship statistics were generated using GraphPad Prism 9.5.1 (GraphPad Software, San Diego, CA, USA) and STATA version 15 (StataCorp, College Station, TX, USA). For continuous data, two group comparisons were using Mann–Whitney. Normality testing and graphical generation were performed using GraphPad Prism 9.5.1 (GraphPad Software, San Diego, CA, USA).

## 3. Results

### 3.1. Identification of Genes Involved in Anticoagulant Metabolism

**Figure 1:**
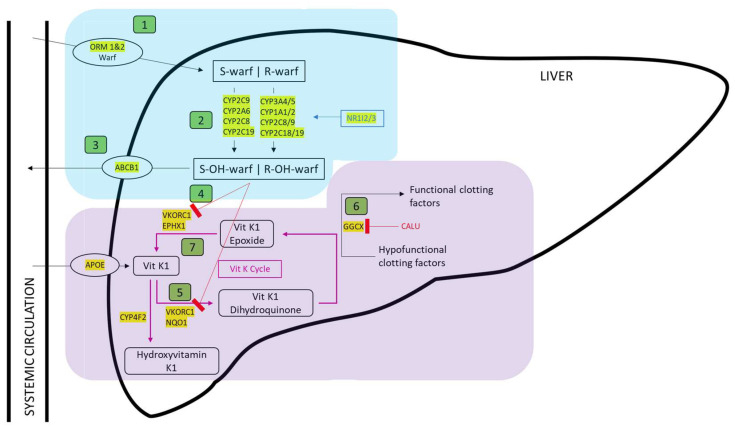
Mechanism of action of warfarin relevant to pharmacogene identification (further details provided in the Data Supplement). Blue box highlights the pharmacokinetic warfarin (warf.) metabolic pathway; purple box the Vitamin K cycles and pharmacodynamic warfarin pathway. Genes encoding participating proteins are highlighted in yellow. Warfarin exerts its anticoagulant effect by inhibiting the functioning of the VKOR enzyme, which results in a reduction in coagulation factors. In detail, warfarin is ① transported to the liver, where it is absorbed and ② metabolised to active metabolites. At this point, some of the drug is ③ eliminated. The remaining active metabolites interfere with the Vitamin K cycle by ④ inhibiting vitamin K epoxide reductase encoded by *VKOR*. Vitamin K1 is ⑤ reduced to Vitamin K1 dihydroquinone, which is an essential cofactor to γ-glutamyl carboxylase. Γ-glutamyl carboxylase ⑥ carboxylates multiple proteins involved in the clotting cascade, including Factor (F)II, FVII, FIX, FX, Protein C, Protein S and Protein Z (encoded by *PROC*, *PROS1* and *PROZ* respectively); proteins involved in bone and tissue modulation such as osteocalcin (encoded by *BGLAP*); circulating matrix Gla protein (encoded by *MGP)*, and apoptosis-related Gas6 (encoded by *GAS6*). The Vitamin K-independent cycle is not included in the diagram. ⑦ The Vitamin K cycle is completed by VKOR and Epoxide Hydrolase 1 (EPHX1) which reduce Vitamin K epoxide to Vitamin K.

**Figure 2:**
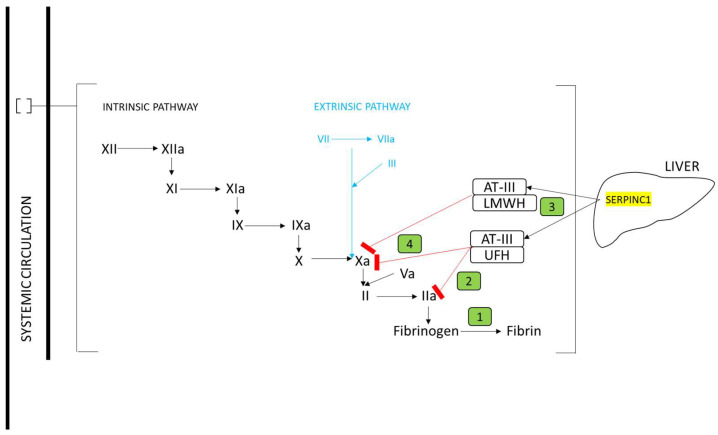
Mechanism of action of heparin relevant to pharmacogene identification (further details provided in the Data Supplement). The coagulation cascade intrinsic (black) and extrinsic (blue) pathways are indicated. Genes encoding participating proteins are highlighted in yellow. ① The conversion of fibrinogen to fibrin is reduced by ② heparin, which inhibits Factors Xa and IIa. Low molecular weight heparin and unfractionated heparin bind to antithrombin III (AT-III encoded by *SERPINC1* and ③ produced in the liver) and ④ enhance inhibition of FXa and FXa plus FIIa, respectively. LMWH: low molecular weight heparin; UFH: unfractionated heparin.

**Figure 3:**
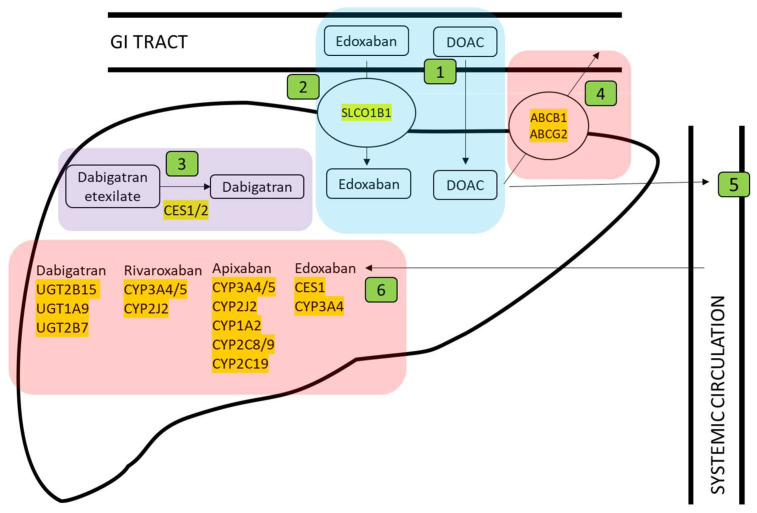
Mechanism of action of direct oral anticoagulants (DOACs) relevant to pharmacogene identification (further details provided in the Data Supplement). DOACs circulating in the blood exert anticoagulant effects by directly inhibiting coagulation factors. Blue box highlights ① DOAC uptake from the gastrointestinal tract following oral ingestion (note: most DOACs are absorbed directly from the gastrointestinal tract into the liver) and ② Edoxaban uptake, which is separately facilitated by an organic anion transporter protein. Purple box highlights ③ activation of dabigatran. Pink boxes highlight DOAC elimination via ④ ATP-binding cassette (ABC) efflux transporters. The remaining DOAC ⑤ circulates in the blood and exerts its anticoagulant effect by directly inhibiting coagulation factors. Eventually, all DOACs will be eliminated via the liver (⑥ highlighted in pink) or kidney.

In total, 44 genes were identified where encoded proteins impacted the pharmacokinetics or pharmacodynamics of warfarin (Figure 1), heparin (Figure 2), or direct oral anticoagulants dabigatran, rivaroxaban, apixaban, edoxaban, and betrixaban (Figure 3).

**Figure 4:**
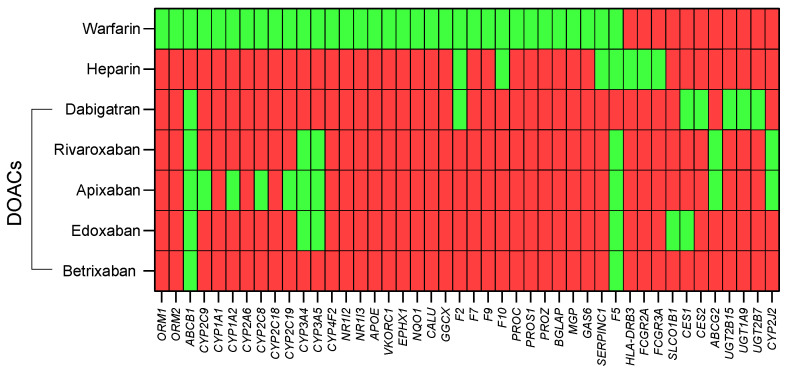
Heat map of the 44 identified pharmacogenes by involvement in mechanisms of action for warfarin, heparin, or selected DOACs (dabigatran, rivaroxaban, apixaban, edoxaban, and betrixaban). Green indicates gene product involved in the mechanism of action; red indicates not involved.

The full list of 44 pharmacogenes is provided in Supplementary Table S2. As shown in Figure 4, individual genes are relevant to more than one drug.

### 3.2. Genomic Location of Genes Involved in Anticoagulant Metabolism

**Figure 5:**
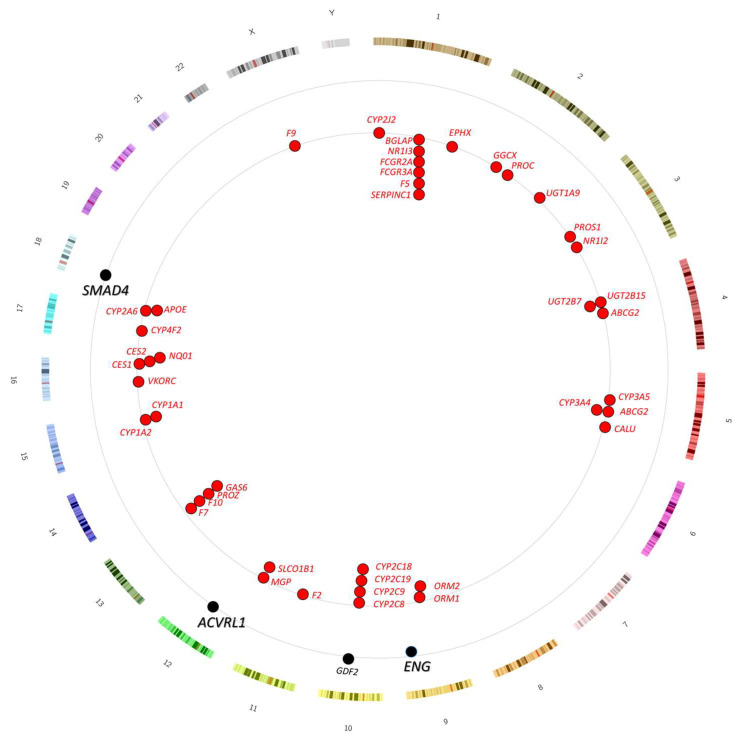
Circus ideogram indicating loci for the HHT genes and the 44 pharmacogenes identified for warfarin, heparin, or DOACs, and the HHT genes. Chromosomes 1–22, X and Y are displayed as an outer ring, HHT genes on the middle ring (black symbols/text), and pharmacogenes on the inner ring (red symbols/text). For precise pharmacogene locations, see Supplementary Table S2.

None of the 44 pharmacogenes genes are located within 1.5 Mb of the major HHT genes *ACVRL1*, *ENG*, or *SMAD4 (*Figure 5, Supplementary Table S2). *ENG* (GRCh38 chr9: 127,815,016–127,854,658) was the closest to one of the pharmacogenes with *ORM2* sited 13.5 Mb away at chr9:114,329,869–114,333,251. However, *ORM2* was almost adjacent (only 75 kb, i.e., 0.075 Mb distant) to the *D9S59* locus that was unlinked to *ENG* in one of the original HHT families used to identify *ENG* as a HHT-causative gene [80]. We concluded that neither HHT gene testing nor familial responses would be likely to predict pharmacogenomic responses to anticoagulants in HHT families.

### 3.3. General Population Variant Burdens

With reference to Figure 1, Figure 2 and Figure 3 predicted loss-of-function (pLOF) alleles, including frameshift indels, nonsense substitutions, splice site, and some missense alleles, would predict higher plasma drug levels for two already actionable pharmacogenes (*VKORC1* for warfarin and *SLCO1B1* for heparin). In addition, for at least 11 further genes, pLOF variants would predict higher plasma drug levels. We, therefore, tested the frequency of pLOF loss-of-function allelic variants.

**Figure 6:**
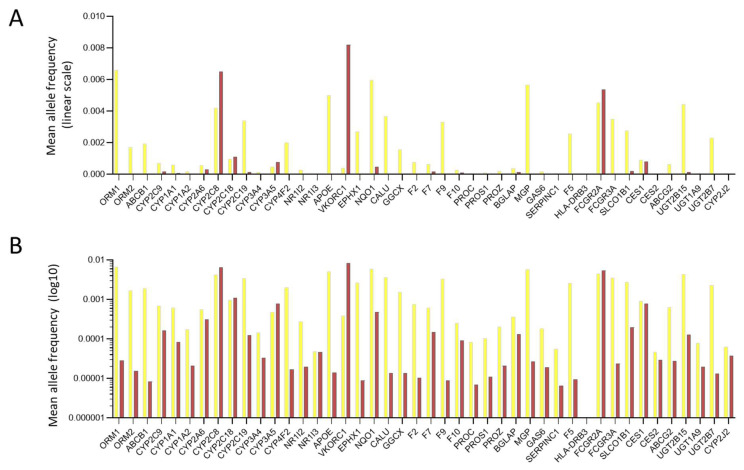
General population variant data in the pharmacogenes. Numeric data were extracted from gnomAD 3.1.2 [77] and have been plotted (**A**) on a linear scale to emphasise relative frequencies; (**B**) on a logarithmic scale to emphasise where pLOF variants are present. Yellow bars indicate missense variants; maroon bars indicate predicted loss-of-function (pLOF) variants.

First, the number of variants within the 44 identified pharmacogenes was examined in the 76,156 participants in gnomAD v3.1.2 [77]. Recruited participants were from diverse backgrounds, including 39,345 Europeans, 20,744 Africans/African Americans, 7647 Latino/Admixed Americans, and 5023 individuals from South or East Asia. In total, there were 9668 different missense substitutions of an amino acid that may be silent but may cause loss-of-function (as for many of the HHT-causal variants in *ACVRL1*, *ENG,* and *SMAD4*) or more rarely, gain-of-function. Separately, there were 1076 different pLOF variants, i.e., frameshift, nonsense, and consensus splice site variants. Variant allele frequencies are displayed in Figure 6 across all genes and were seen in all ethnicities. Importantly, within the gnomAD sample of 76,156 people, this approximately translated to 1 in 7.9 individuals having a missense substitution and 1 in 71 a predicted loss of function allele.

Adjusting for gene length, *VKORC1*, an actionable gene for warfarin prescription [71,72,73], had the greatest number of missense and pLOF variants per coding region nucleotide (nt) at 0.34 missense variants per nucleotide and 0.045 pLOF variants per nucleotide. *F9* encoding Factor 9 had the fewest at 0.051 missense variants/nt and 0.014 pLOF variants/nt. The number of pLOF variants per coding region nucleotide passed normality testing using all four of Anderson–Darling, D’Agostino and Pearson, Shapiro–Wilk, and Kolmogorov-Smirnov tests (all *p* values > 0.09) in support of random origin and maintenance. Missense variants did not pass normality testing using any of the four tests (all *p* values > 0.097, with *VKORC1* and *F9* being outliers).

### 3.4. HHT Population Variant Burdens

**Figure 7:**
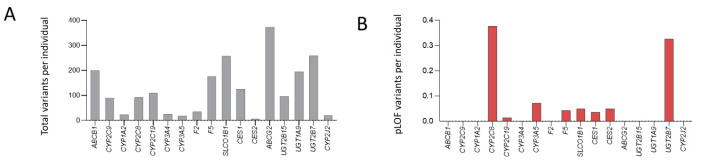
HHT patient variant prevalence in the 17 pharmacogenes for DOACs. Variant prevalence data was ascertained through the 141 HHT patients recruited to the 100,000 Genomes Project, ref. [78] expressed as variants per individual, and plotted for (**A**) total and (**B**) pLOF variants.

While there was no reason to expect HHT patients to have differing proportions of variants in these genes, we took the opportunity to examine variant frequencies in the 100,000 Genomes Project-recruited HHT population. Mindful of the change in general medical practice away from warfarin and heparin requiring efficacy assessments and/or injections, towards direct oral anticoagulants that do not, we focused on the 17 genes relevant to DOAC mechanisms of action. As shown in Figure 7, within the modestly sized (N = 141) HHT population recruited to the 100,000 Genomes Project, variants were identified in all genes, with pLOF variants identified in eight genes.

**Figure 8:**
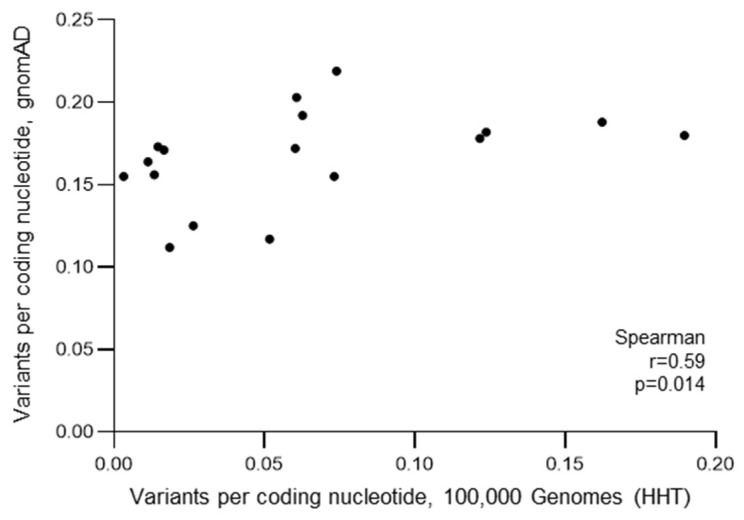
Correlation between variant numbers ascertained in the general population through the Genome Aggregation Database (gnomAD) v3.1.2 (general population) [77] and the hereditary haemorrhagic telangiectasia (HHT) population recruited to the 100,000 Genomes Project [78]. Numeric data were extracted from the respective sources, and variants per coding nucleotide calculated and plotted as described in the Methods.

Despite the differing methods of ascertainment, genome alignments, and stringency metrics, there was a direct correlation between the number of variants per coding sequence nucleotide in the HHT cohort and that identified in the gnomAD general population. In other words, the number of variants per coding sequence nucleotide in the HHT cohort increased as the number of variants per coding sequence nucleotide in the general population increased (*p* = 0.014, Figure 8). Further, for pLOF variants where the impact would be confidently predicted, there were between 0 and 53 per gene across the 141 HHT patients, representing an average of 0–0.38 per patient per gene. Overall, across all 17 genes implicated in DOAC mechanisms of action, the mean number of pLOF variants per HHT patient recruited to the 100,000 Genomes Project was 0.96 (standard deviation 0.11).

## 4. Discussion

We have shown that across a series of 44 genes where gene products influence pharmacokinetics or pharmacodynamics of major, currently used anticoagulants, at least 1 in 7 individuals can be expected to have a missense substitution, and more than 1 in 70 to have a loss-of-function variant. This includeds drug metabolism pharmacogenes in which heterozygous loss-of-function alleles, along with other pharmacogenes-encoding drug transporters and receptors, predict higher drug levels. The variants were also identified in people with HHT, where there is a narrower therapeutic window due to abnormal vasculature. Thus, while it is not possible to predict bleeding tendency based on familial HHT gene variants or phenotypes, knowledge of pharmacogenetic variants may allow predictions facilitating individualised anticoagulant prescriptions. Conversely, for non-genotyped populations, chance differences in the prevalence of these variants could result in skewed results of less general applicability across HHT than previously thought.

The main study limitations were the absence of functional data in the participants with the gene variants and the lack of ethnic diversity in the HHT patients recruited to the 100,000 Genomes Project (though more ethnically diverse genome datasets were examined through gnomAD). However, the main study strength is to alert the field of the presence of these variants and their potential importance to prescribing.

In terms of implications for practice, the first element is relevance to prescribing anticoagulants in HHT without pharmacogenomic data, as this is the current situation. Previous work has shown marked variability in anticoagulant tolerance in terms of bleeding in HHT [39,40,55,56,57,58,59,60,61,62], and the current findings are in keeping with this. Previous work has also shown that individuals with HHT who are unable to tolerate one particular anticoagulant due to excessive bleeding are able to tolerate other agents [55,57]. Again, the current findings are in keeping with this. Given the narrow therapeutic window and tendency to higher thrombotic rates in HHT [38,39,40], there is value in being able to monitor efficacy directly through laboratory assays (e.g., international normalized ratio (INR) for warfarin [81] or activated partial thromboplastin time (APTT) for heparin [82]) to ensure the patient is anticoagulated to the correct degree. For warfarin, different loading regimens can be employed, and a more conservative low-dose approach, such as the Crowther protocol [83], may be preferred above a rapid loading schedule, such as recommended by Tait [84].

Where pharmacogenetic testing is available, validated variants can be translated into personalised prescribing by employing internationally recognised guidelines such as those found on PharmGKB [73]. That said, of the anticoagulants examined in the current manuscript, only warfarin has clinically established pharmacogenomic prescribing guidelines [85,86,87,88]. Even amongst these well-established warfarin guidelines, there is a lack of consensus with differing approaches to dose alterations arising from clinically actionable pharmacogenomic variants. Notably, with alternatives such as efficacy monitoring through the INR [81] and wider use of DOACs [89,90], there may not be a push to harmonise the warfarin guidance.

For DOACs, which are now the main oral anticoagulants in clinical use [91,92], the data are in the arena of “newly discovered variants”, where PharmGKB recommends determining the level of evidence for the impact a variant would have on whether a dosing amendment is needed [93]. The effect of a loss-of-function variant can be inferred using the principles from the American College of Medical Genetics and Association for Molecular Pathology [94] for “very important pharmacogenes” in which the drug–gene association has been strongly established. However, for variants where drug–gene associations have not been made, studies examining the association between pharmacogenetic variants and drug response would need to be performed. This is the case for all DOACs where pharmacogenomic testing is not yet used in clinical practice [95].

There is a trend towards greater adoption of pharmacogenomic testing in mainstream clinical practice to improve safety and efficacy. For example, England’s National Health Service commissions a limited number of individual drug–gene pairs [96], and the goal is to move towards pre-emptive panel-based testing across a wide range of drug–gene pairs [97], providing clinically actionable genetic information at the point of prescribing. The recent multicentre randomised control trial, PREPARE, demonstrated the success of this approach by testing for 50 variants in 12 pharmacogenes resulting in a significantly lower number of adverse drug events [68]. Subsequently, the PROGRESS programme is assessing the feasibility of introducing NHS-wide genetic testing to guide prescription in common practice [98,99]. Although economic panel-based arrays are proposed as the first step to introduce pharmacogenomics into common practice [65,96,97,98,99], as shown by our study, whole genome sequencing facilitates the identification of novel clinically relevant loss-of-function variants, which would otherwise go undetected. As such, as sequencing costs fall, this approach may become more commonplace.

It must be recognised that pharmacogenomics will be implemented at different rates in different healthcare structures. So, taken together, what conclusions can be drawn from the current HHT study? First, as guideline-emphasised, therapeutic anticoagulation is not contraindicated in HHT [7]. If no personalised genomic data are available, drug treatment can still be personalised using demographic and clinical data, remembering the HHT-specific observational studies summarised in Supplementary Table S1. Given that DOACs have so many advantages for easier prescription (though they are more difficult to monitor and reverse), this is the area where pharmacogenomics may be of most potential importance to HHT and other states with narrower therapeutic windows for anticoagulation. For people with HHT, the significant bleeding risk posed by anticoagulation, and their higher incidence of VTE, together with nosebleeds providing less severe manifestations of excessive bleeding, represent a strong argument in favour of further study in the population.

In order to address some of the study limitations, future work could use functional data to validate pharmacogene variants. In addition, associations between pharmacogene variants and drug response could be measured. Although prospective clinical trials may not be ethical in this population, retrospective analyses could be performed; alternatively, pharmacogenomic data from unaffected individuals could be extrapolated to HHT patients. It will also be important to educate patients about the benefits of pharmacogenomic testing to increase awareness and acceptance of this approach, and of anticoagulation when clinically indicated. Ultimately, since most indications for anticoagulants are in the emergency setting, prior understanding of individual drug risk profiles would enhance patient safety.

## 5. Conclusions

High proportions of the HHT population carry DNA variants that predict particular anticoagulants will carry a higher risk of haemorrhage. In view of their narrow therapeutic window and the usually urgent nature of anticoagulant prescribing, we encourage the development of pre-emptive pharmacogenomic testing alongside HHT gene testing. More generally, the HHT population is well placed to be at the vanguard of personalised prescribing.

## Data Availability

Data supporting reported results can be found in the Data Supplement. Primary data from the 100,000 Genomes Project, which are held in a secure Research Environment, are available to registered users. Please see https://www.genomicsengland.co.uk/research/academic (accessed on 11 December 2023) for further information.

## Supplementary Materials

The following supporting information can be downloaded at https://www.mdpi.com/2077-0383/12/24/7710/s1: Supplementary Methods; Supplementary Table S1: Previous HHT Anticoagulation Studies; Supplementary Table S2: Pharmacogenes and location; Supplementary References [39,40,55,56,57,58,59,60,100,101,102,103,104,105,106,107,108,109,110,111,112,113,114,115,116,117,118,119,120,121,122,123,124].
