# Supplementary material for: Pharmacogenomic Considerations for Anticoagulant Prescription in Patients with Hereditary Haemorrhagic Telangiectasia
Source: J Clin Med. 2023 Dec 15;12(24):7710. doi: 10.3390/jcm12247710 (PMC10744266; doi:10.3390/jcm12247710)
Supplement: Supplementary file 1 [file jcm-12-07710-s001.zip › jcm-2746832-supplementary.pdf]

Pharmacogenomic considerations for anticoagulant prescription in patients with hereditary haemorrhagic telangiectasia

DATA SUPPLEMENT

Supplementary Text 2

Biochemical pathways generated from literature search 2

Warfarin 2

Heparin 2

Direct Oral Anticoagulants [DOACs] 3

Supplementary Table S1: HHT anticoagulant studies 4

Supplementary Table S2: Pharmacogenes and genomic location 5

Supplementary References 7

Supplementary Text

Biochemical pathways generated from literature search

Warfarin

(see Figure 1)

Warfarin is administered as a racemic mixture consisting of an S- enantiomer and the less potent R-enantiomer. Two alpha-1-acid glycoproteins that bind and transport warfarin in the blood, where it is then taken up by the liver,[100,101] are orosomucoid 1 (or alpha-1-acid glycoprotein 1) and orosomucoid 2 (alpha-1-acid glycoprotein 2), and these are encoded by *ORM1*, and *ORM2* respectively. Warfarin is primarily metabolised by the cytochrome P450 (CYP) 2C9 (CYP2C9) and CYP2C19 enzymes, with additional involvement of the CYP2A6, CYP3A4, CYP3A5, CYP1A1, CYP1A2, CYP2C8, and CYP2C18 enzymes. There is evidence that some warfarin elimination occurs via the P-glycoproteins transporters in the liver, which are encoded by *ABCB1*] (ATP binding cassette subfamily B member 1).[102,103] Many of the cytochrome P450 enzymes are inducible when warfarin is co-administered with other drugs, such as rifampicin and phenobarbital, with the pregnane X receptor gene (encoded by *NR1I2*) and constitutive androstane receptor gene (encoded by *NR1I3*, Nuclear receptor subfamily 1 group I member 3) playing a role in the mechanism of induction.[104–106]

Warfarin acts by interfering with the recycling of Vitamin K by inhibiting the vitamin K epoxide reductase (VKOR) enzyme, which is involved in the conversion of vitamin K epoxide into a reduced form.[107,108] *APOE* encodes the Apolipoprotein E receptor that is involved the uptake of Vitamin K into the liver.[109,110] Dietary quinone Vitamin K is reduced to the dihydroquinone form by VKOR (encoded by *VKORC1*)and NAD(P)H (encoded by *NQO1*, NAD(P)H quinone dehydrogenase 1).[111,112] A side pathway exits whereby Vitamin K is converted to hydroxyvitamin K by CYP4F2.[113] Vitamin K dihydroquinone is an essential cofactor for the enzyme γ-glutamylcarboxylase (GGCX) which then catalyses the post-translational-glutamyl carboxylation of the clotting factors II (prothrombin), VII, IX, and X; Proteins C, S, and Z; proteins involved in bone and tissue modulation (bone gamma-carboxyglutamate protein encoded by *BGLAP*; matrix Gla protein encoded by *MGP*, and apoptosis-related growth arrest specific 6 encoded by *GAS6.*[107,108,114]) Calumenin (*CALU*) inhibits GGXC which prevents carboxylation of proteins.[115]. The Vitamin K cycle is completed by VKOR and epoxide hydrolase 1 (*EPHX1*) which reduce Vitamin K epoxide to Vitamin K [116]. It should also be noted that anti-thrombin III (encoded by *SERPINC1*) and Factor V are non-vitamin K-dependent clotting proteins that could potentially effect warfarin disposition. Anti-thrombin III inhibits Factors II, IX, X, XI and XII, and may interfere with warfarin induction when in a hypercoagulable state, whilst Factor V meditates the conversion of prothrombin to thrombin with warfarin intake reducing this conversion.[102,117]

Heparin

(see Figure 2)

Heparin is administered intravenously allowing it to bypass the gastrointestinal (GI) tract and directly affect its targets.[118] Heparin is available in two forms, unfractionated heparin (UFH) and low molecular weight heparin (LMWH). UFHs and LMWHs differ in chain length and molecular weight which results in slightly variable anticoagulation mechanisms. Heparins exert their antithrombotic effect by interfering with the coagulation cascade, specifically interfering with the conversion of prothrombin (Factor II) to thrombin (Factor IIa). Both UFHs and LMWHs bind antithrombin III (encoded by SERPINC1) which enhances inhibition of Factor Xa, reducing the conversion of prothrombin to

thrombin. Additionally, the UFH-34 antithrombin III complex is further able to bind and inhibit thrombin directly, due to its longer chain length. LMWHs are unable to achieve this and therefore act mostly via Factor Xa inhibition [119]. Factor Va mediates the conversion of prothrombin to thrombin and can influence the response to heparin therapy. Final elimination of heparin occurs via renal clearance [120].

Direct Oral Anticoagulants [DOACs]

(see Figure 3)

Most DOACs share a common mode of action. The DOAC passes through the GI tract where it is absorbed into the liver. Hepatic uptake, of edoxaban specifically, involves organic anion transporter protein 1B1 (OATP1B1), encoded by SLCO1B1 (solute carrier organic anion transporter family member 1B1).[121]. Once in the liver, the inactive dabigatran precursor is converted to its active form by the primary carboxylesterase, CES1 and, to a lesser degree, CES2 (encoded by *CES1* and *CES2*, respectively). [122,123] Other DOACs are already in their active form. The DOACs then go on to circulate in the blood.[124] If the DOACs do not enter the systemic circulation, they are transported back into the GI tract, via the ABC (ATP-binding cassette) efflux transporters encoded by *ABCB1* and *ABCG2*, for excretion [56,57]. The remaining drug circulates the blood and exerts its anticoagulant effect.

DOACs work by directly inhibiting coagulation factors, such as Factor IIa (thrombin) in the case of dabigatran, or Factor Xa (Stuart factor) in the case of rivaroxaban, apixaban, edoxaban, and betrixaban.[55] Eventually all DOACs will be eliminated. Dabigatran is metabolised by UDP-glucuronosyltransferase enzymes, UGT2B15, UGT1A9, and UGT2B7.[39] Rivaroxaban and apixaban are metabolised by CYP3A4/5 and CYP2J2, with apixaban having an additional minimal participation of CYP1A2, CYP2C8, CYP2C9, and CYP2C19.[122,58] Edoxaban is metabolised by CES1 with a small contribution by CYP3A4/5.[55] Unlike the other DOAC factor Xa inhibitors, betrixaban has a less than 1% contribution by CYP450 enzymes and is excreted unchanged.[40,55]

Supplementary Table S1: HHT anticoagulant studies

Authors, year	Source of data	Location	Design	Anticoagulant treatment episodes	Proportion prophylactic dose †	Heparin	VKAs	DOACS	Other‡	Premature Discontinuation rate
Edwards et al, 2012 [56]	Toronto HHT Database (1997-2009)	Canada, single centre	Retrospective	40	7(17%)*	20*	20	0	0	20% including antiplatelets
Devlin et al, 2013 [57]	International HHT survey	North America>Europe	Patient Survey	150	unclear	93*	55	2		Not specified
Shovlin et al, 2019 [55]	8 European VASCERN HHT Reference Centres	Denmark, France, Germany, Italy, the Netherlands and the UK	Retrospective	32	0%	0	0	32	0	27.5% (Rivaroxaban 35.7%; Apixaban 20%)
Riera-Mestre et al, 2019 [39]	RIETE ('Registro Informatizado Enfermedad TrombóEmbolica') registry (2009 to 2019)	24 countries (205 centres)	Retrospective	23	0%	13	8	1	1	Not specified
Gaetani et al, 2020 [58]	Gemelli Hospital HHT Registry (2016 to 2018)	Italy (single centre)	Retrospective	19	4(21%)*	6*	4	5	4	20%
Tentoni et al, 2021 [40]	Hospital Italiano de Buenos Aires HHT institutional Registry (2006 to 2019)	Buenos Aires, Argentina	Retrospective	28	0%	9	19	0	0	25%
Virk et al, 2023 [59]	MGH/ Brigham Research Patient Data Registry (1996-2022)	USA- 5 hospitals	Retrospective	87	0%	26	35	25	1	44%
Grobost et al, 2023 [60]	French National HHT Registry (2010 to 2021)	France- national network	Retrospective	108	29%	25*	37	44*	unclear	34.6%

Table highlights the small study size, retrospective or survey nature, and where combined analysis of therapeutic and lower prophylactic doses. Heparin refers to low molecular weight heparins and unfractionated heparin (therapeutic and prophylactic doses where indicated†/*); VKAs: Vitamin K antagonists such as warfarin and acenocoumarol (therapeutic dosage only); DOACs, direct oral anticoagulants such as apixaban, rivaroxaban,edoxaban and dabigatran (therapeutic and prophylactic doses where indicated†/*); Other: alternate measures such as Vena Cava Filter, fondaparinux or omitted information.

Supplementary Table S2: Pharmacogenes and genomic location

Gene	Genome Build	Position	Size (nt)	Exon Count	Strand	Anticoagulant with gene involvement
ORM1	hg38	chr9:114,323,098-114,326,479	3.382	6	+	Warfarin
ORM2	hg38	chr9:114,329,869-114,333,251	3,383	6	+	Warfarin
ABCB1	hg38	chr7:87,503,017-87,600,884	97,868	28	-	Warfarin, DOAC
CYP2C9	hg38	chr10:94,938,658-94,990,091	51,434	9	+	Warfarin, DOAC
CYP1A1	hg38	chr15:74,719,542-74,725,528	5,987	7	-	Warfarin
CYP1A2	hg38	chr15:74,748,845-74,756,607	7,763	7	+	Warfarin, DOAC
CYP2A6	hg38	chr19:40,843,541-40,850,447	6,907	9	-	Warfarin
CYP2C8	hg38	chr10:95,036,772-95,069,497	32,726	9	-	Warfarin, DOAC
CYP2C18	hg38	chr10:94,683,729-94,736,190	52.462	9	+	Warfarin
CYP2C19	hg38	chr10:94,762,681-94,855,547	92,867	9	+	Warfarin, DOAC
CYP3A4	hg38	chr7:99,756,967-99,784,184	27,218	13	-	Warfarin, DOAC
CYP3A5	hg38	chr7:99,648,194-99,679,996	31,803	13	-	Warfarin, DOAC
CYP4F2	hg38	chr19:15,878,023-15,898,074	20,052	13	-	Warfarin
NR1I2	hg38	chr3:119,782,101-119,818,487	36,387	9	+	Warfarin
NR1I3	hg38	chr1:161,229,669-161,238,203	8,535	9	-	Warfarin
APOE	hg38	chr19:44,905,796-44,909,393	3,598	4	+	Warfarin
VKORC1	hg38	chr16:31,090,854-31,094,797	3,944	3	-	Warfarin
EPHX1	hg38	chr1:225,810,124-225,845,563	35,440	9	+	Warfarin
NQO1	hg38	chr16:69,709,401-69,726,560	17,160	6	-	Warfarin
CALU	hg38	chr7:128,739,359-128,773,400	34,042	7	+	Warfarin
GGCX	hg38	chr2:85,544,720-85,561,493	16,774	15	-	Warfarin
F2	hg38	chr11:46,719,213-46,739,506	20,294	14	+	Warfarin, heparin
F7	hg38	chr13:113,105,791-113,120,685	14,895	8	+	Warfarin
F9	hg38	chrX:139,530,739-139,563,459	32,721	8	+	Warfarin
F10	hg38	chr13:113,122,799-113,149,529	26,731	8	+	Warfarin, heparin
PROC	hg38	chr2:127,418,427-127,429,242	10,816	9	+	Warfarin
PROS1	hg38	chr3:93,873,051-93,973,896	100,846	15	-	Warfarin
PROZ	hg38	chr13:113,158,648-113,172,386	13,739	8	+	Warfarin

…./Gene	Genome Build	Position	Size (nt)	Exon Count	Strand	Anticoagulant with gene involvement
BGLAP	hg38	chr1:156,242,184-156,243,317	1,134	4	+	Warfarin
MGP	hg38	chr12:14,880,864-14,885,854	4,991	4	-	Warfarin …/
GAS6	hg38	chr13:113,820,549-113,864,076	43,528	15	-	Warfarin
SERPINC1	hg38	chr1:173,903,800-173,917,327	13,528	7	-	Warfarin, heparin
F5	hg38	chr1:169,511,951-169,586,481	74,531	25	-	Warfarin, heparin
FCGR2A	hg38	chr1:161,505,457-161,519,829	14,373	7	+	Heparin
FCGR3A	hg38	chr1:161,541,759-161,549,818	8,060	5	-	Heparin
SLCO1B1	hg38	chr12:21,131,194-21,239,796	108,603	15	+	DOAC
CES1	hg38	chr16:55,802,851-55,833,096	30,246	14	-	DOAC
CES2	hg38	chr16:66,935,516-66,945,096	9,581	12	+	DOAC
ABCG2	hg38	chr4:88,090,269-88,158,639	68,371	16	-	DOAC
UGT2B15	hg38	chr4:68,646,597-68,670,652	24,056	6	-	DOAC
UGT1A9	hg38	chr2:233,671,898-233,773,300	101,403	5	+	DOAC
UGT2B7	hg38	chr4:69,096,474-69,112,987	16,514	6	+	DOAC
CYP2J2	hg38	chr1:59,893,308-59,926,773	33,466	9	-	DOAC

Nt: nucleotide; hg38: GRCh38. Note *ENG* is located at GRCh38 chr9:127,815,016-127,854,658; *ACVRL1* at chr12:51907504-51923361, *SMAD4* at chr18:51030213-51085042, and *GDF2* at chr10:47,322,454-47,327,588. Also not shown is *HLA-DRB3* (relevant to heparin, Figure 2) which is at chr6(ALT_REF_LOCI_6):3,715,352-3,728,489.

Supplementary References

39. Riera-Mestre A, Mora-Luján JM, Trujillo-Santos J, Del Toro J, Nieto JA, Pedrajas JM, López-Reyes R, Soler S, Ballaz A, Cerdà P, Monreal M; RIETE Investigators. Natural history of patients with venous thromboembolism and hereditary hemorrhagic telangiectasia. Findings from the RIETE registry. Orphanet J Rare Dis. 2019 Aug 9;14(1):196.

40. Tentoni N, Lapidus MI, Peuchot VA, Vazquez FJ, Serra MM. Bleeding events during anticoagulation in patients with hereditary hemorrhagic telangiectasia. Thromb Res. 2021 Jan;197:109-111.

55. Shovlin CL, Millar CM, Droege F, Kjeldsen A, Manfredi G, Suppressa P, Ugolini S, Coote N, Fialla AD, Geisthoff U, Lenato GM, Mager HJ, Pagella F, Post MC, Sabbà C, Sure U, Torring PM, Dupuis-Girod S, Buscarini E; VASCERN-HHT. Safety of direct oral anticoagulants in patients with hereditary hemorrhagic telangiectasia. Orphanet J Rare Dis. 2019 Aug 28;14(1):210.

56. Edwards CP, Shehata N, Faughnan ME. Hereditary hemorrhagic telangiectasia patients can tolerate anticoagulation. Ann Hematol. 2012;91(12):1959-68.

57. Devlin HL, Hosman AE, Shovlin CL. Antiplatelet and anticoagulant agents in hereditary hemorrhagic telangiectasia. N Engl J Med. 2013;368(9):876-8

58. Gaetani E, Agostini F, Giarretta I, Porfidia A, Di Martino L, Gasbarrini A, Pola R, On Behalf Of The Multidisciplinary Gemelli Hospital Group For Hht. Antithrombotic Therapy in Hereditary Hemorrhagic Telangiectasia: Real-World Data from the Gemelli Hospital HHT Registry. J Clin Med. 2020 Jun 2;9(6):1699

59. Virk ZM, Zhang E, Rodriguez-Lopez J, Witkin A, Wong AK, Luther J, Lin AE, Ning M, Grabowski E, Holbrook EH, Al-Samkari H. Safety, tolerability, and effectiveness of anticoagulation and antiplatelet therapy in hereditary hemorrhagic telangiectasia. J Thromb Haemost. 2023 Jan;21(1):26-36.

60. Grobost V, Hammi S, Pereira B, Guilhem A, Duffau P, Seguier J, Parrot A, Gautier G, Alric L, Kerjouan M, Le Guillou X, Simon D, Chaussavoine L, Rondeau-Lutz M, Leguy-Seguin V, Delagrange L, Lavigne C, Maillard H, Dupuis-Girod S; French HHT group. Antiplatelet and anticoagulant therapies in hereditary hemorrhagic telangiectasia: A large French cohort study (RETROPLACOTEL). Thromb Res. 2023 Sep;229:107-113.

100. Nakagawa T, Kishino S, Itoh S, Sugawara M, Miyazaki K. Differential binding of disopyramide and warfarin enantiomers to human alpha(1)-acid glycoprotein variants. Br J Clin Pharmacol. 2003;56(6):664–9.

101. Otagiri M, Maruyama T, Imai T, Suenaga A, Imamura Y. A comparative study of the interaction of warfarin with human α1-acid glycoprotein and human albumin. Journal of Pharmacy and Pharmacology. 1987;39(6):416–20.

102. Wadelius M, Pirmohamed M. Pharmacogenetics of warfarin: current status and future challenges. The Pharmacogenomics Journal. 2007;7(2):99–111.

103. Wadelius M, Sörlin K, Wallerman O, Karlsson J, Yue QY, Magnusson PKE, et al. Warfarin sensitivity related to CYP2C9, CYP3A5, ABCB1 (MDR1) and other factors. The Pharmacogenomics Journal. 2003;4(1):40–8.

104. Assenat E, Gerbal-Chaloin S, Larrey D, Saric J, Fabre JM, Maurel P, et al. Interleukin 1beta inhibits CAR-induced expression of hepatic genes involved in drug and bilirubin clearance. Hepatology. 2004;40(4):951–60.

105. Lehmann JM, McKee DD, Watson MA, Willson TM, Moore JT, Kliewer SA. The human orphan nuclear receptor PXR is activated by compounds that regulate CYP3A4 gene expression and cause drug interactions. Journal of Clinical Investigation. 1998;102(5):1016–23. /pmc/articles/PMC508967/?report=abstract

106. Chen Y, Ferguson SS, Negishi M, Goldstein JA. Induction of human CYP2C9 by rifampicin, hyperforin, and phenobarbital is mediated by the pregnane X receptor. J Pharmacol Exp Ther. 2004;308(2):495–501.

107. Jonas DE, Mcleod HL. Genetic and clinical factors relating to warfarin dosing. Trends Pharmacol Sci. 2009;30(7):375–86.

108. Moualla H, Garcia D. Vitamin K Antagonists – Current Concepts and Challenges. Thromb Res. 2011;128(3):210–5.

109. Kohlmeier M, Salomon A, Saupe J, Shearer MJ. Transport of vitamin K to bone in humans. J Nutr. 1996;126(4 Suppl):1192S-6S.

110. Yu W ying, Sun X, Wadelius M, Huang L, Peng C, Ma W Le, et al. Influence of APOE Gene Polymorphism on Interindividual and Interethnic Warfarin Dosage

Requirement: A Systematic Review and Meta-Analysis. Cardiovasc Ther. 2016;34(5):297–307.

111. Tian L, Xiao P, Zhou B, Chen Y, Kang L, Wang Q, et al. Influence of NQO1 Polymorphisms on Warfarin Maintenance Dose: A Systematic Review and Meta-Analysis (rs1800566 and rs10517). Cardiovasc Ther. 2021;2021:5534946.

112. Mishima E, Ito J, Wu Z, Nakamura T, Wahida A, Doll S, et al. A non-canonical vitamin K cycle is a potent ferroptosis suppressor. Nature. 2022;608(7924):778–83.

113. McDonald MG, Rieder MJ, Nakano M, Hsia CK, Rettie AE. CYP4F2 Is a Vitamin K1 Oxidase: An Explanation for Altered Warfarin Dose in Carriers of the V433M Variant. Mol Pharmacol. 2009;75(6):1337–46.

114. Danziger J. Vitamin K-dependent proteins, warfarin, and vascular calcification. Clinical Journal of the American Society of Nephrology. 2008;3(5):1504–10.

115. Vecsler M, Loebstein R, Almog S, Kurnik D, Goldman B, Halkin H, et al. Combined genetic profiles of components and regulators of the vitamin K-dependent γ-carboxylation system affect individual sensitivity to warfarin. Thromb Haemost. 2006;95(02):205–11.

116. Loebstein R, Vecsler M, Kurnik D, Austerweil N, Gak E, Halkin H, et al. Common Genetic Variants of Microsomal Epoxide Hydrolase Affect Warfarin Dose Requirements Beyond the Effect of Cytochrome P450 2C9. Clin Pharmacol Ther. 2005;77(5):365–72.

117. Larsen TB, Lassen JF, Dahler-Eriksen BS, Petersen PH, Brandslund I. Effect of anticoagulant therapy on the hypercoagulable state in patients carrying the factor V Arg506Gln mutation. Thromb Res. 1998;92(4):157–62.

118. Franchini M, Liumbruno GM, Bonfanti C, Lippi G. The evolution of anticoagulant therapy. Blood Transfus. 2016;14(2):175–84.

119. Oduah EI, Linhardt RJ, Sharfstein ST.. Heparin: Past, Present, and Future. Pharmaceuticals. 2016;9(3):38.

120. Alridha AMA, Al-Gburi KM, Abbood SK. A review of pharmacogenetics of anticoagulant therapy: Heparins, rivaroxaban, apixaban, and dabigatran. Medical Journal of Babylon. 2023;19(3):332–40.

121. Vandell AG, Lee J, Shi M, Rubets I, Brown KS, Walker JR. An integrated pharmacokinetic/pharmacogenomic analysis of ABCB1 and SLCO1B1 polymorphisms on edoxaban exposure. The Pharmacogenomics Journal. 2016;18(1):153–9.

122. Shnayder NA, Petrova MM, Shesternya PA, Savinova A V., Bochanova EN, Zimnitskaya O V., et al. Using Pharmacogenetics of Direct Oral Anticoagulants to Predict Changes in Their Pharmacokinetics and the Risk of Adverse Drug Reactions. Biomedicines. 2021;9(5):451.

123. Merali Z, Ross S, Paré G. The pharmacogenetics of carboxylesterases: CES1 and CES2 genetic variants and their clinical effect. Drug Metabol Drug Interact. 2014;29(3):143–51.

124. Tseng AS, Patel RD, Quist HE, Kekic A, Maddux JT, Grilli CB, et al. Clinical Review of the Pharmacogenomics of Direct Oral Anticoagulants. Cardiovasc Drugs Ther. 2018;32(1):121–6.
